# Vitamin D and adverse pathologic features in patients undergoing radical prostatectomy in the contemporary magnetic resonance imaging‐guided management era

**DOI:** 10.1002/bco2.70261

**Published:** 2026-08-04

**Authors:** Sarita Chitjaroen, Kantima Jongjitaree, Varat Woranisarakul, Siros Jitpraphai, Tawatchai Taweemonkongsap, Sunai Leewansangtong, Sittiporn Srinualnad, Thitipat Hansomwong

**Affiliations:** ^1^ Division of Urology, Department of Surgery, Faculty of Medicine Siriraj Hospital Mahidol University Bangkok Thailand

**Keywords:** adverse pathologic features, magnetic resonance imaging, prostate cancer, radical prostatectomy, vitamin D

## Abstract

**Objective:**

This study aimed to evaluate the association between serum 25‐hydroxyvitamin D (25‐OH D) levels and adverse pathologic features in patients undergoing radical prostatectomy (RP) in the contemporary magnetic resonance imaging (MRI)‐guided management era.

**Methods:**

This retrospective study included patients who underwent RP between January 2022 and December 2024 at a single academic centre. Preoperative MRI was routinely incorporated into staging and treatment planning. Serum 25‐OH D levels measured within 3 months prior to surgery were analysed. Vitamin D insufficiency was defined as 20–29.99 ng/mL and deficiency as <20 ng/mL. Adverse pathologic features included International Society of Urological Pathology (ISUP) Grade Group 4 or 5, extraprostatic extension (EPE), seminal vesicle invasion (SVI) and pelvic lymph node metastasis. Associations were evaluated using multivariable logistic regression analysis.

**Results:**

A total of 217 patients were included. Preoperative MRI was performed in 96.0% of patients and MRI‐targeted biopsy in 78.3%. Adverse pathologic features were identified in 66.4% of patients. Vitamin D insufficiency and deficiency were present in 67.3% and 17.5%, respectively. Mean serum 25‐OH D levels did not differ significantly between patients with and without adverse pathology (28.19 ± 8.41 ng/mL vs. 27.46 ± 9.26 ng/mL, *p* = 0.55). In multivariable analysis, body mass index and adverse MRI findings were independently associated with adverse pathology, whereas serum 25‐OH D levels were not. An exploratory subgroup analysis demonstrated an association between serum 25‐OH D levels and EPE in very‐high‐risk patients.

**Conclusion:**

Serum 25‐OH D levels were not independently associated with adverse pathologic features following RP in this contemporary MRI‐guided management cohort. While established clinical and radiologic factors remain the primary predictors of adverse pathology, further studies are needed to clarify the role of vitamin D in long‐term oncologic and functional outcomes.

## 1 INTRODUCTION

Prostate cancer is a biologically heterogeneous disease, and adverse pathologic features following radical prostatectomy (RP)—such as high International Society of Urological Pathology (ISUP) grade, extraprostatic extension (EPE), seminal vesicle invasion (SVI) and pelvic lymph node metastasis—are strongly associated with disease progression and prostate cancer‐specific mortality.^1^, ^2^, ^3^, ^4^, ^5^, ^6^ Accurate preoperative identification of aggressive disease remains essential for treatment selection, surgical planning, and prognostication.

Vitamin D insufficiency is common among patients with prostate cancer.^7^, ^8^, ^9^, ^10^ Experimental studies have suggested that vitamin D may exert antitumour effects through antiproliferative, prodifferentiation and immunomodulatory mechanisms.^11^, ^12^, ^13^, ^14^ Several clinical studies have reported an association between low serum 25‐hydroxyvitamin D (25‐OH D) levels and adverse pathologic features following RP and prostate biopsy.^15^, ^16^ However, findings have been inconsistent, and most prior investigations were conducted in Western populations and in the pre‐multiparametric magnetic resonance imaging (MRI) era, before the routine integration of MRI into prostate cancer management.

The widespread adoption of multiparametric MRI and MRI‐guided biopsy has substantially improved preoperative risk stratification by reducing disease understaging and enhancing the detection of clinically significant cancer.^17^, ^18^ Consequently, associations between biomarkers and pathology observed in earlier cohorts may not directly translate to contemporary settings, where MRI‐guided management has refined preoperative risk stratification. Furthermore, data from Asian populations remain limited. Therefore, we aimed to reassess the predictive value of preoperative serum 25‐OH D levels for adverse pathologic features in Thai patients undergoing RP in the contemporary MRI‐guided management era.

## 2 MATERIALS AND METHODS

### 2.1 Study design and population

This retrospective cohort study included consecutive patients with histologically confirmed prostate adenocarcinoma who underwent RP between January 2022 and December 2024 at a single academic centre in Bangkok, Thailand. Preoperative multiparametric MRI, interpreted using Prostate Imaging‐Reporting and Data System (PI‐RADS) version 2.1, was routinely incorporated into staging and treatment planning during the study period.

Patients who received neoadjuvant androgen deprivation therapy or radiotherapy prior to surgery were excluded. Patients without available serum 25‐OH D measurements within 3 months before RP were also excluded. The three‐month interval was selected to reflect the biological half‐life of circulating 25‐OH D, which is approximately 2–3 weeks.^19^

### 2.2 Ethical approval

The study protocol was approved by the Institutional Review Board of our institution (COA no. Si 971/2024). All procedures were conducted in accordance with the Declaration of Helsinki and institutional research policies. Given the retrospective study design, the requirement for informed consent was waived by the Institutional Review Board. The study was conducted and reported in accordance with the STROBE (Strengthening the Reporting of Observational Studies in Epidemiology) guidelines.

### 2.3 Sample size calculation

Sample size estimation was based on the study by Nyame et al., which reported a significant difference in mean serum 25‐OH D levels between patients with and without adverse pathologic features following RP. Assuming an expected mean difference of 5 ng/mL in serum 25‐OH D levels between groups and a standard deviation of 10 ng/mL, a conservative effect size was applied.

Using a two‐sided *α* level of 0.05, 90% statistical power and a 1:3 allocation ratio between patients with and without adverse pathologic features—reflecting the distribution observed in prior institutional cohorts^20^—the required sample size was calculated using nQuery Advisor software. The final cohort size met these requirements, supporting adequate power to detect clinically meaningful differences in serum 25‐OH D levels.

### 2.4 Definitions

Adverse pathologic features were defined as ISUP Grade Group 4 or 5, EPE, SVI or pelvic lymph node metastasis on final RP pathology. Pelvic lymph node dissection was performed in all patients.

MRI adverse features were defined as radiologic evidence of EPE, SVI, or pelvic lymph node metastasis on preoperative multiparametric MRI.

Neutrophil‐to‐lymphocyte ratio (NLR) and platelet‐to‐lymphocyte ratio (PLR) were calculated from preoperative complete blood counts.

### 2.5 Outcomes

The primary outcomes were (1) the prevalence of vitamin D insufficiency and deficiency among patients undergoing RP and (2) the association between preoperative serum 25‐OH D levels and adverse pathologic features on final RP pathology.

Exploratory analyses were performed to evaluate associations between serum 25‐OH D levels and individual pathologic components, including EPE, within predefined risk subgroups based on the National Comprehensive Cancer Network (NCCN) classification. These analyses were considered hypothesis‐generating and were not powered for definitive inference.

### 2.6 Vitamin D assessment

Serum 25‐OH D levels were obtained from routine preoperative laboratory testing. Vitamin D deficiency was defined as <20 ng/mL and insufficiency as 20–29.99 ng/mL, in accordance with established clinical guidelines.^21^

### 2.7 Statistical analysis

Continuous variables were summarized as mean ± standard deviation or median with interquartile range, as appropriate. Normality was assessed using the Shapiro–Wilk test and visual inspection of histograms. Comparison between groups was performed using Student's *t*‐test or the Mann–Whitney *U* test for continuous variables and the chi‐square test or Fisher's exact test for categorical variables, as appropriate.

Univariable logistic regression analysis was performed to identify factors associated with adverse pathologic features. Variables that were clinically relevant or demonstrated univariable *p* < 0.10 were entered into multivariable models.

Receiver operating characteristic (ROC) analysis was conducted in exploratory subgroup analyses to evaluate the discriminatory ability of serum 25‐OH D levels for predicting EPE. The optimal cutoff value was determined using the Youden index. All statistical tests were two‐sided, and *p* < 0.05 was considered statistically significant. All statistical analyses were performed using IBM SPSS Statistics version 28.0 (IBM Corp., Armonk, NY, USA).

## 3 RESULTS

### 3.1 Patient characteristics and vitamin D status

A total of 217 patients undergoing RP were included. The mean age was 68.12 ± 6.42 years, and the median PSA level was 8.76 ng/mL (IQR 6.08–14.1). According to the NCCN 2024 classification, 7.4% of patients were categorized as very low or low risk, 17.5% favourable intermediate risk, 10.6% unfavourable intermediate risk, 42.4% high risk and 22.1% very high risk.

Adverse pathologic features were identified in 144 patients (66.4%). Compared with patients without adverse pathology, those with adverse pathology had significantly higher PSA levels, PSA density and body mass index (BMI). MRI adverse features were more frequently observed in the adverse pathology group (38.7% vs. 13.9%, *p* < 0.001).

Preoperatively, 209 patients (96.0%) underwent multiparametric MRI, and 170 patients (78.3%) underwent MRI‐transrectal ultrasound fusion‐guided biopsy, which included both targeted and systematic sampling. MRI‐guided biopsy was more common in patients with adverse pathology, whereas transrectal ultrasound‐guided biopsy was more frequent among patients without adverse pathology (*p* = 0.01) (Table 1). NLR and PLR did not differ significantly between groups.

**TABLE 1:** Baseline characteristics and pathologic findings stratified by adverse pathologic features.

Variable	Adverse pathology, *n* = 144 (66.4%)	No adverse pathology, *n* = 73 (33.6%)	*p‐*value
Age (years, mean ± SD)	67.98 ± 6.44	68.40 ± 6.42	0.65
BMI (kg/m^2^, mean ± SD)	25.2 ± 3.42	23.98 ± 3.62	0.01
PSA (ng/mL, median [IQR])	9.75 (6.18–17.71)	7.41 (5.72–11.57)	0.005
PSAD (ng/mL^2^, median [IQR])	0.29 (0.19–0.57)	0.20 (0.13–0.28)	<0.001
5‐ARI use, *n* (%)	14 (9.7)	3 (4.1)	0.15
Smoking, *n* (%)	3 (2.1)	1 (1.4)	0.59
Preoperative MRI, *n* (%)	137 (95.1)	72 (98.6)	0.27
MRI adverse features, *n* (%)	53 (38.7)	10 (13.9)	<0.001
Biopsy method, *n* (%)			
TRUS	38 (26.4)	6 (8.2)	0.01
MRI‐fusion biopsy	103 (71.5)	67 (91.8)	
Others (e.g., TURP, HoLEP)	3 (2.1)	0 (0)	
Serum 25‐OH D (ng/mL, mean ± SD)	28.19 ± 8.41	27.46 ± 9.26	0.56
Vitamin D supplementation, *n* (%)	11 (7.6)	7 (9.6)	0.62
NLR (median [IQR])	1.97 (1.46–2.84)	1.85 (1.39–2.41)	0.24
PLR (median [IQR])	118.18 (89.5–148.71)	109.14 (82.67–137.71)	0.07

*Note*: PSAD = PSA density, calculated using prostate volume measured on MRI; 5‐ARI = 5‐alpha‐reductase inhibitor; MRI adverse features were defined as the presence of EPE SVI or pelvic lymph node metastasis on MRI; TURP = transurethral resection of the prostate; HoLEP = holmium laser enucleation of the prostate; TRUS = transrectal ultrasound‐guided prostate biopsy‐performed as a systematic non‐targeted biopsy; MRI‐fusion biopsy = MRI/ultrasound fusion‐guided prostate biopsy comprising targeted biopsy and a systematic10–12 cores biopsy; NLR = neutrophil‐to‐lymphocyte ratio; PLR = platelet‐to‐lymphocyte ratio.

The mean serum 25‐OH D level in the overall cohort was 27.94 ± 8.60 ng/mL. Vitamin D insufficiency and deficiency were present in 67.3% and 17.5%, respectively. Mean serum 25‐OH D levels did not differ significantly between patients with and without adverse pathologic features (28.19 ± 8.41 vs. 27.46 ± 9.26 ng/mL; *p* = 0.55) (Table 1).

### 3.2 Association between serum 25‐OH D levels and adverse pathologic features

Stratification into serum 25‐OH D categories (<20, 20–29.99, 30–39.99 and ≥40 ng/mL) demonstrated no significant associations with ISUP Grade Group 4–5, EPE, SVI, pelvic lymph node metastasis or intraductal carcinoma of the prostate (IDCP) (Table 2). MRI adverse features were more frequently observed in patients with serum 25‐OH D levels ≥40 ng/mL (*p* = 0.025); however, these levels were not significantly associated with adverse features in the final prostatectomy pathology.

**TABLE 2:** Adverse pathologic and MRI findings across serum 25‐hydroxyvitamin D categories in patients undergoing radical prostatectomy.

Pathologic feature	<20, *n* = 38 (17.5%)	20–29.99, *n* = 108 (49.8%)	30–39.99, *n* = 50 (23%)	≥40, *n* = 21 (9.7%)	*P*‐value
ISUP Grade Group					
1–3	26 (68.4)	74 (68.5)	33 (66)	12 (57.1)	
4–5	12 (31.6)	34 (31.5)	17 (34)	9 (42.9)	0.78
EPE	20 (52.6)	68 (63)	30 (60)	12 (57.1)	0.72
SVI	7 (18.4)	20 (18.5)	9 (18)	3 (14.3)	0.97
Pelvic lymph node metastasis	3 (7.9)	3 (2.8)	3 (6)	0 (0)	0.36
IDCP	10 (26.3)	21 (19.4)	7 (14)	3 (14.3)	0.48
MRI adverse features	10 (27.8)	28 (26.7)	13 (27.1)	12 (60)	0.025

*Note*: MRI adverse features were defined as the presence of EPE, SVI or pelvic lymph node metastasis.

Abbreviations: EPE, extraprostatic extension; IDCP, intraductal carcinoma of the prostate; ISUP, International Society of Urological Pathology; SVI, seminal vesicle invasion.

Using an exploratory cutoff of 22 ng/mL, corresponding to the mean serum 25‐OH D levels reported for the adverse pathology group by Nyame et al.,^15^ no significant differences in adverse pathologic features were observed between groups (Table 3).

**TABLE 3:** Association between serum 25‐hydroxyvitamin D levels and adverse pathologic features using the 22 ng/mL threshold proposed by Nyame et al.

Pathologic feature	<22 ng/mL, *n* = 55 (25.3%)	≥22 ng/mL, *n* = 162 (74.7%)	*P‐*value
ISUP Grade Group 4 or 5, *n* (%)	14 (25.5)	58 (35.8)	0.16
Extraprostatic extension (EPE), *n* (%)	30 (54.5)	100 (61.7)	0.35
Seminal vesicle invasion (SVI), *n* (%)	8 (14.5)	31 (19.1)	0.44
Pelvic lymph node metastasis, *n* (%)	3 (5.5)	6 (3.7)	0.70
MRI adverse features, *n* (%)	12 (23.1)	51 (32.5)	0.20
PSA persistence, *n* (%)	3 (6.4)	16 (11.8)	0.41
Need for adjuvant therapy, *n* (%)	9 (16.7)	29 (18.4)	0.78

*Note*: PSA persistence was defined as a PSA level >0.1 at 3 months after surgery; the need for adjuvant therapy was defined as the initiation of androgen deprivation therapy or radiation therapy within 3 months after surgery.

In univariable logistic regression analysis, higher BMI, PSA, PSA density and MRI adverse features were associated with adverse pathologic outcomes. In multivariable analysis, BMI (odds ratio [OR] 1.11, 95% confidence interval [CI] 1.01–1.22; *p* = 0.03) and MRI adverse features (OR 3.52, 95% CI 1.60–7.78; *p* = 0.002) remained independently associated with adverse pathology. Serum 25‐OH D levels were not associated with adverse pathologic features in either univariable or multivariable models (Table 4).

**TABLE 4:** Logistic regression analysis of factors associated with adverse pathologic features.

Variable	Univariate OR (95% CI)	*P*‐value	Multivariate OR (95% CI)	*p*‐value
Age	0.99 (0.95–1.04)	0.65	—	—
BMI	1.11 (1.02–1.22)	0.02	1.11 (1.01–1.22)	0.03
PSA	1.03 (1.00–1.06)	0.05	1.02 (0.99–1.05)	0.24
5‐ARI use	2.51 (0.70–9.04)	0.16	3.14 (0.83–11.87)	0.09
MRI adverse features	3.91 (1.85–8.29)	<0.001	3.52 (1.60–7.78)	0.002
Type of surgery: LRP	1.14 (0.10–13.31)	0.92	—	—
Type of surgery: RALRP	0.92 (0.08–10.41)	0.95	—	—
Serum 25‐OH D level	1.01 (0.98–1.04)	0.56	1.00 (0.97–1.04)	0.86
Vitamin D supplementation	0.78 (0.29–2.10)	0.62	—	—
NLR	1.01 (0.93–1.20)	0.44	—	—
PLR	1.00 (0.99–1.01)	0.20	1.00 (0.99–1.01)	0.28

Abbreviations: 5‐ARI, 5‐alpha‐reductase inhibitor; BMI, body mass index; LRP, laparoscopic radical prostatectomy; NLR, neutrophil‐to‐lymphocyte ratio; PLR, platelet‐to‐lymphocyte ratio; RALRP, robotic assisted laparoscopic radical prostatectomy.

### 3.3 Exploratory subgroup and ROC analyses

In subgroup analyses stratified by NCCN risk category, an association between serum 25‐OH D levels and EPE was observed in the very‐high‐risk group (OR 0.88, 95% CI 0.77–0.99; *p* = 0.035). No significant associations were identified in other risk groups (Table 5).

**TABLE 5:** Association between serum 25‐hydroxyvitamin D levels and extraprostatic extension across NCCN 2024 risk groups.

NCCN 2024 risk group	Serum 25‐OH (ng/mL, mean ± SD)		*p*‐value	Odds ratio (95% CI)	*p‐*value
	No EPE	EPE			
Very low/low, *n* = 16 (7.4%)	28.22 ± 14.43 (*n* = 13)	22.30 ± 5.01 (*n* = 3)	0.25	0.95 (0.81–1.11)	0.49
Favourable intermediate, *n* = 38 (17.5%)	25.81 ± 7.37 (*n* = 33)	23.96 ± 5.62 (*n* = 5)	0.30	0.96 (0.83–1.12)	0.59
Unfavourable intermediate, *n* = 23 (10.6%)	31.13 ± 9.34 (*n* = 19)	24.80 ± 3.46 (*n* = 4)	0.10	0.89 (0.75–1.06)	0.21
High, *n* = 92 (42.4%)	25.74 ± 7.55 (*n* = 16)	28.74 ± 8.78 (*n* = 76)	0.10	1.05 (0.97–1.13)	0.21
Very high, *n* = 48 (22.1%)	35.42 ± 7.14 (*n* = 6)	27.58 ± 7.62 (*n* = 42)	0.011	0.88 (0.77, 0.99)	0.035

Abbreviations: EPE, extraprostatic extension; NCCN risk group, National Comprehensive Cancer Network risk group of prostate cancer.

**FIGURE 1:**
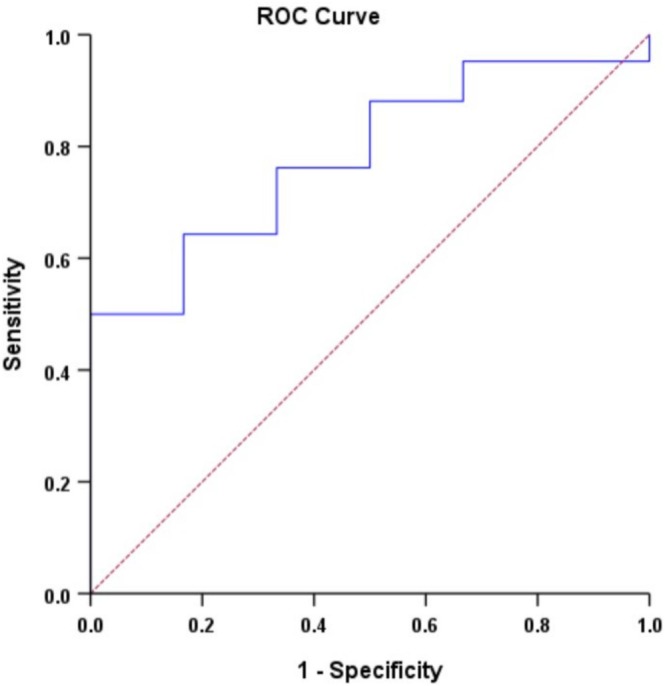
Receiver operating characteristic curve for serum 25‐hydroxyvitamin D levels in predicting extraprostatic extension among patients in the very‐high‐risk group.

Within the very‐high‐risk subgroup, ROC analysis demonstrated an area under the curve (AUC) of 0.782 (95% CI 0.62–0.94; *p* = 0.027) for serum 25‐OH D levels in predicting EPE. The optimal cutoff value was 26.05 ng/mL, yielding a sensitivity of 50.0% and a specificity of 100% (Figure 1).

### 3.4 Pathologic concordance and risk group outcomes

Comparison of Gleason scores between biopsy and RP pathology demonstrated concordance in 63% of cases, upgrading in 26.5%, and downgrading in 10.5%.

Among lower‐risk patients (including very low, low, and intermediate risk), the proportions with EPE, SVI and pelvic lymph node metastasis on final pathology were 15%, 1% and 0%, respectively.

## 4 DISCUSSION

In this contemporary MRI‐guided management cohort of Thai patients undergoing RP, serum 25‐OH D levels were not independently associated with adverse pathologic features, including high ISUP grade, EPE, SVI or pelvic lymph node metastasis. Although vitamin D insufficiency (67.3%) and deficiency (17.5%) were common, serum 25‐OH D levels did not predict adverse pathology after adjustment for established clinical and radiologic variables. In contrast, a high serum 25‐OH D level (≥40 ng/mL) was significantly associated with adverse radiologic features on MRI.

The prevalence of vitamin D insufficiency and deficiency in our cohort exceeded that reported in the general Thai male population (32.6% and 1.9%, respectively),^22^ despite Thailand's near‐equatorial location and abundant year‐round sunlight.^23^ This discrepancy suggests that patients with prostate cancer may represent a metabolically distinct population with reduced outdoor activity, comorbid conditions, or systemic inflammatory states contributing to lower serum 25‐OH D levels. However, increased prevalence does not necessarily translate into predictive value for tumour aggressiveness.

Nyame et al. reported one of the few studies examining serum 25‐OH D and prostatectomy pathology and demonstrated lower vitamin D levels in patients with adverse pathology in a predominantly Western, pre‐MRI era cohort.^15^ In contrast, we did not observe such an association, even when applying the same exploratory cutoff value. Differences in diagnostic era, ethnicity, lifestyle and environmental exposure may partly explain these divergent findings. Moreover, our study revealed a novel finding: A high serum 25‐OH D level was significantly associated with adverse MRI features, despite its lack of association with adverse pathologic features following RP. While a large meta‐analysis^24^ previously linked high serum 25‐OH D levels (specifically around 40 ng/mL) to an increased incidence of prostate cancer, it is critical to note that its findings pertained to cancer incidence rather than tumour aggressiveness—our primary focus. Therefore, future studies are required to explore this discordance between radiologic adverse features and final pathologic adverse features in patients with higher serum 25‐OH D levels.

Importantly, our study was conducted in the contemporary MRI‐guided management era. The widespread integration of multiparametric MRI and MRI‐guided biopsy has substantially reduced pathologic understaging and improved concordance between biopsy and final RP pathology.^17^, ^18^, ^25^, ^26^, ^27^, ^28^ In earlier cohorts relying primarily on systematic transrectal ultrasound‐guided biopsy, understaging may have confounded associations between biomarkers and final pathology. Within a modern imaging‐integrated diagnostic pathway, serum 25‐OH D levels did not provide incremental predictive value beyond established clinical and MRI‐based risk factors.

BMI remained independently associated with adverse pathologic features, although the effect size was modest (OR 1.11 per kg/m^2^ increase). Obesity has been linked to adverse prostate cancer characteristics^29^ through mechanisms involving chronic low‐grade inflammation, insulin resistance and altered sex hormone metabolism.^30^, ^31^ Given that higher BMI is associated with lower circulating vitamin D levels,^32^, ^33^ this raises the possibility that metabolic factors may partially confound previously reported associations between vitamin D status and tumour aggressiveness. In the present cohort, however, serum 25‐OH D levels were not independently associated with adverse pathology after adjustment for BMI, suggesting that broader metabolic factors may play a more prominent role than vitamin D alone in this surgical population.

A Japanese cohort study by Sawada et al. similarly reported no significant association between serum 25‐OH D levels and prostate cancer risk in a nested case–control analysis.^34^ Although conducted in the premultiparametric MRI era and focusing on cancer incidence rather than surgical pathology, this finding aligns with the absence of association observed in our cohort. Ethnicity‐dependent polymorphisms in the vitamin D receptor (VDR) have been linked to prostate cancer susceptibility,^35^ suggesting that population‐specific genetic factors may influence vitamin D‐related tumour biology.

Exploratory subgroup analysis identified an association between serum 25‐OH D levels and EPE in very‐high‐risk disease. ROC analysis suggested discriminatory ability and identified a data‐derived cutoff of 26.05 ng/mL. While this finding may reflect a potential relationship between vitamin D status and local tumour extension in advanced disease, this cutoff should be interpreted cautiously given the exploratory design and limited subgroup size. Specifically, within this very‐high‐risk subgroup, 42 patients had EPE, whereas only 6 did not. This marked imbalance, particularly the small number of patients without EPE, limits the precision of the specificity estimate and the immediate clinical applicability of the ROC findings.

Vitamin D supplementation has been investigated in selected prostate cancer populations, including patients undergoing active surveillance, with reports of reduced positive biopsy cores and slower PSA kinetics.^36^, ^37^ Observational studies have also suggested an association between supplementation and reduced overall cancer mortality.^38^ Although vitamin D supplementation was not assessed in the present study and our endpoint was limited to surgical pathology, these data suggest that vitamin D may influence broader oncologic or systemic outcomes beyond adverse pathologic features alone.

Systemic inflammatory markers (NLR and PLR) were not associated with adverse pathologic features in this cohort. This finding is consistent with reports suggesting that inflammatory indices may have greater prognostic relevance in advanced or metastatic disease rather than in a localized surgically treated population.^39^, ^40^, ^41^, ^42^, ^43^

This study has several limitations. Its retrospective, single‐centre design may limit generalizability. Serum 25‐OH D was measured at a single time point without adjustment for or detailed assessment of supplementation. Although adequately powered for the primary analysis, subgroup and ROC analyses were exploratory. Strengths include a contemporary MRI‐era cohort, uniform surgical pathology endpoints and a focus on an underrepresented Southeast Asian population.

## 5 CONCLUSION

In this contemporary MRI‐guided management cohort of Thai patients undergoing RP, preoperative serum 25‐OH D levels were not independently associated with adverse pathologic features. Although vitamin D insufficiency was more prevalent in patients with prostate cancer than in the general Thai male population, vitamin D status did not provide additional predictive value beyond established clinical and imaging‐based risk factors.

An exploratory association between serum 25‐OH D levels and EPE was observed in very‐high‐risk disease and warrants cautious interpretation. These findings underscore the importance of diagnostic context when evaluating biomarker–pathology relationships and support further investigation into the broader biologic and prognostic roles of vitamin D in advanced prostate cancer.

## AUTHOR CONTRIBUTIONS

**Sarita Chitjaroen:** Conceptualization; data curation; investigation; formal analysis; writing—original draft. **Kantima Jongjitaree:** Data curation; investigation; writing—review and editing. **Varat Woranisarakul:** Investigation; resources; writing—review and editing. **Siros Jitpraphai:** Investigation; resources; writing—review and editing. **Tawatchai Taweemonkongsap:** Resources; supervision; writing—review and editing. **Sunai Leewansangtong:** Resources; supervision; writing—review and editing. **Sittiporn Srinualnad:** Conceptualization; supervision; writing—review and editing. **Thitipat Hansomwong:** Conceptualization; methodology; formal analysis; validation; supervision; project administration; writing—original draft; writing—review and editing.

## CONFLICT OF INTEREST STATEMENT

The authors declare that they have no known competing financial interests or personal relationships that could have appeared to influence the work reported in this paper.

## DECLARATION OF GENERATIVE AI IN SCIENTIFIC WRITING

During the preparation of this manuscript, generative artificial intelligence tools were used to assist with language editing and improvement of grammar and clarity. The authors critically reviewed and edited all content generated by these tools and take full responsibility for the integrity, accuracy, and originality of the manuscript.

## CONSENT TO PARTICIPATE

The requirement for informed consent was waived by the Siriraj Institutional Review Board (SIRB) due to the retrospective nature of the study and the use of de‐identified patient data.

## CONSENT FOR PUBLICATION

Not applicable.

## Data Availability

The data from this study are not deposited in a publicly accessible repository. However, the data are available upon reasonable request for academic purposes. Please contact the corresponding author (T.H.) for further details.
